# Acromioclavicular Reconstruction Using the Lockdown Technique: A Case Series and Systematic Review

**DOI:** 10.3390/jcm14124046

**Published:** 2025-06-07

**Authors:** Krisztian Kovacs, Szilárd Váncsa, Zsolt Abonyi-Tóth, Peter Hegyi, Gergely Soos, Kalman Rabai, Tibor Bogosi, Gyorgy Kocsis

**Affiliations:** 1 Department of Orthopaedics, Semmelweis University, 1082 Budapest, Hungary; kovacs.krisztian.balazs@semmelweis.hu; 2 Centre for Translational Medicine, Semmelweis University, 1082 Budapest, Hungary; vancsaszilard@gmail.com (S.V.); abonyi-toth.zsolt@univet.hu (Z.A.-T.); hegyi2009@gmail.com (P.H.); sooska99@gmail.com (G.S.); 3 Institute for Translational Medicine, Medical School, University of Pécs, 7622 Pécs, Hungary; 4 Institute of Pancreatic Diseases, Semmelweis University, 1082 Budapest, Hungary; 5 Department of Biostatistics, University of Veterinary Medicine, 1078 Budapest, Hungary; 6 Manninger Jenő Traumatology Center, 1081 Budapest, Hungary; rabai.kalman@gmail.com; 7 Military Hospital Medical Centre, Hungarian Defense Forces, 1134 Budapest, Hungary; bogosi.dr@gmail.com

**Keywords:** acromioclavicular joint, shoulder injuries, lockdown technique, complications, shoulder scores, case series

## Abstract

**Background:** Acromioclavicular (AC) joint dislocations are frequent, especially kocsisamong young male athletes. While over 150 surgical techniques exist, consensus on optimal treatment—particularly for Rockwood type III injuries—remains elusive. This study evaluates the Lockdown procedure’s efficacy, safety, and patient satisfaction, a synthetic ligament technique for AC joint stabilization. **Methods:** A multicenter prospective study was conducted on 39 patients across three Hungarian hospitals (2018–2023). Outcomes included shoulder function, pain levels, and complication rates, with subgroup analysis of acute (≤3 weeks) versus chronic (>3 weeks) cases. A systematic review of nine studies (205 cases) was also performed to assess broader outcomes and complications. **Results:** Significant improvements were observed in functional scores (OSS, Constant, DASH, SST, ASES, Nottingham, Imitani) and pain reduction, especially in acute cases with no prior shoulder surgery. The mean patient age was 38.9 ± 12.68 years, with a 24.5-month average follow-up. OSS improvement between acute and chronic cases was 14.96 (95% CI: 6.45–23.47; *p* = 0.0017). Complications (30.8%) occurred in eleven patients, mainly minor infections; implant failure necessitated revision in 5.1%. The systematic review reported a 34.6% complication rate (predominantly minor complications, like asymptomatic subluxation −16%) and 5.4% implant removal due to failure. A meta-analysis was not feasible due to data heterogeneity. **Conclusions:** The Lockdown procedure significantly enhances shoulder function and reduces pain, particularly in acute dislocations. However, the procedure showed a moderate complication rate, underscoring the need for careful patient selection and postoperative management.

## 1. Introduction

Acromioclavicular (AC) joint dislocations are common injuries, which occur in 3.5–12% of all injuries in the shoulder girdle [1,2]. They were initially classified by Tossy and Allmann, with modifications by Green and Rockwood, who categorized AC injuries into six types [3]. There is no consensus on treatment, but low-energy injuries (Rockwood type I and II) are typically managed conservatively with a harness or a sling [4].

Severe injuries (Rockwood type III and above) generally require surgery, with over 150 surgical techniques available. For separating these techniques, we can distinguish between AC fixation, coracoclavicular (CC) fixation, resection of the distal clavicle with coracoacromial ligament transfer, and ligament reconstruction using non-synthetic grafts or synthetic grafts such as Lockdown itself. However, the treatment of type III injuries is still controversial in the literature [4,5,6]. Gold standardization of techniques is an important tool to help surgeons, which is missing and needed in this condition. Other authors also recognized this gap, and protocols were created for a systematic review (SR) and meta-analysis (MA), but without the optimal data they are not feasible [7].

**Figure 1:**
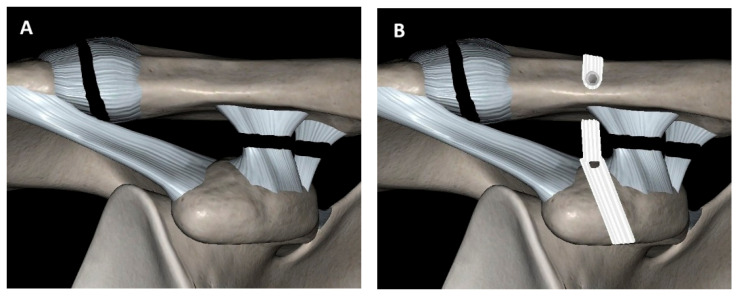
Lockdown technique (**A**) acromioclavicular joint (ACJ) dislocation and coracoclavicular (CC) ligament rupture. (**B**) Lockdown synthetic graft looped around the coracoid process: ACJ stabilization.

Among the numerous surgical options, the Lockdown procedure shows promising results [8]. Originating from Nottingham and previously known as Surgilig, it has been in use since 2001. In contrast to other rigid fixation devices—such as Bosworth screws, plates, and Kirschner wires—the Lockdown system offers the advantage of eliminating the need for implant removal. Additionally, its synthetic band also promotes tissue ingrowth [9]. The technique employs a double-braided polyester ligament to functionally replace the coracoclavicular (CC) ligaments and restore the anatomical position of the clavicle, thereby re-establishing dynamic stability [10] (Figure 1). Despite the long presence of this technique, the reported studies are low in quality and contain many gaps, although studies report low failure rates and high functional outcomes with Surgilig [8].

Our study first aimed to evaluate the efficacy, safety, and patient satisfaction of the Lockdown technique in treating Rockwood type III and above AC injuries across three Hungarian centers, and, second, to perform a systematic review and meta-analysis to compare our findings with the existing literature. We hypothesized that the Lockdown technique is more effective in acute and primary AC injuries than chronic and revisional cases.

## 2. Materials and Methods

### 2.1. Methods of the Case-Series Study

The first part of this study is a multicentric prospective data collection with prospective follow-up of patients treated in three Hungarian hospitals (two traumatology centers and one university orthopedic department). The study received ethical approval from the Semmelweis University Regional and Institutional Committee of Science and Research Ethics (approval ID: BMEÜ/2832-3/2022/EKU). All procedures followed the PROCESS 2020 (Preferred Reporting of Case Series in Surgery) guidelines (Table S1) [11]. All procedures were made by three senior surgeons across the centers who followed the same surgical technique.

#### 2.1.1. Patient Selection

This multicentric prospective cohort study enrolled 39 patients who underwent Lockdown synthetic ligament surgery between January 2018 and January 2023. Eligible patients were over 16 years of age, with isolated injury of the AC joint, and free from mental illness affecting postoperative rehabilitation. Patients with no data or who were unable to give consent were excluded. Enrollment occurred in three centers (at the Department of Orthopaedics, Semmelweis University, Dr. Manninger Jenő Traumatology Center, and the Department of Traumatology, Hungarian Defence Forces Medical Centre).

#### 2.1.2. Data Extraction, Data Types, and Measures

Trained physicians, who did not participate in the operations at any center, extracted data from hospital electronic records to ensure accuracy. Follow-up data were collected through medical records, outpatient visits, and telephone interviews. The sample size was determined by recording the number of patients. Data included sex (male or female), age at surgery, shoulder function scores, classification of surgeries as primary or revision, and injury severity according to the Rockwood classification (types III, IV, V). Information documented also included surgical complications, discharge (fluid released from the wound) status, trauma details, and any reoperations. Patients were categorized as acute (trauma within three weeks) or chronic (trauma more than three weeks prior). Our nonsignificant findings were evaluated in the context of minimal clinically important difference (MCID) values reported in the literature [12].

#### 2.1.3. Surgical Technique

Patients were positioned in a beach chair position, and a vertical incision was made over the clavicle. A lasso was formed around the coracoid process to reposition the clavicle to measure the implant. The selected implant was inserted using the lasso and length gauge loop. After fixation of the lasso, the clavicle was stabilized with a 3.5-mm cortical screw placed through a pre-drilled hole in the clavicle itself. The wound was closed in layers. A detailed description is provided in the article by Jeon et al. [13].

#### 2.1.4. Outcomes

The study measured several outcomes both preoperatively and postoperatively. These included the Oxford Shoulder Score, the IMITANI Score, the CONSTANT Score, and the UCLA (University of California-Los Angeles) Shoulder Score. Additionally, the DASH Score (Disabilities of the Arm, Shoulder, and Hand), the SST Percentage (Simple Shoulder Test), the ASES Score (American Shoulder and Elbow Surgeons), and the Numerical Rating Scale (NRS, on a scale between 0 to 10) were evaluated.

#### 2.1.5. Statistical Methods

Statistical analysis was carried out in “R” (R Core Team 2022, v4.3.1) and MESS 1.3.1. using data structured in Excel. Two-sample *t*-tests were used for subgroup comparisons. Boxplots were utilized to represent each outcome based on baseline characteristics. All *p*-values were two-sided, and the significance level was 5%.

### 2.2. Methods of the Systematic Review

We conducted our systematic review and meta-analysis according to PRISMA 2020 guidelines (Table S2) and the Cochrane Handbook [14,15]. Only case series and case reports with individual measurements were included. The study protocol was registered on PROSPERO (CRD42024578461).

#### 2.2.1. Information Sources and Search Strategy

On 10 August 2024, we conducted a systematic search using PubMed (Medline), Cochrane Central Library, Embase, Scopus, and Web of Science. We also included an additional report from other reviews. The search terms used were “acromioclavicular AND (lockdown OR surgilig OR synthetic)”, with no filters applied.

#### 2.2.2. Selection Process

We used Endnote v21.0 (Clarivate Analytics) for reference management. After removing duplicates, two independent reviewers (KK and GS) selected studies based on titles and abstracts, achieving a Cohen’s kappa of 0.99. The full-text selection was performed by the same reviewers with a Cohen’s kappa of 0.91. A third reviewer resolved any disagreements.

#### 2.2.3. Eligibility Criteria

Eligible studies reported outcomes of the Lockdown (or Surgilig) technique for acute or chronic acromioclavicular injuries. Exclusion criteria included studies involving cadavers or nonhuman specimens, multiple injuries, conference abstracts, editorials, biomechanical studies, descriptions of techniques, non-Lockdown procedures, systematic reviews, or meta-analyses. We included case series and clinical trials on Rockwood III or higher injuries assessed by shoulder scores.

#### 2.2.4. Data Collection Process and Data Items

Two authors (KK, GS) collected data from eligible articles, with a third author reviewing them to minimize extraction errors. Data extracted included the first author, DOI, country, publication year, number of cases, gender ratio, mean age at surgery, duration of follow-up, injury classification, time to surgery, pre- and postoperative shoulder scores, pain, deformity, duration of physiotherapy, and postoperative complications. Implant-related complications were identified when reoperation led to implant removal.

#### 2.2.5. Evaluation of the Studies and Study Risk of Bias Assessment

Due to the low number of studies, heterogeneous data, and incomplete outcome reporting, we decided not to perform a meta-analysis of the extracted data. We summarized the results of the systematic review in a narrative form and presented data in summary tables. The risk of bias assessment was performed by two authors (KK, GS). Each article was assessed using the JBI critical appraisal tool for case series and the ROBINS-I tool [16,17].

## 3. Results

### 3.1. Case-Series Study

#### 3.1.1. Case-Series Study, Basic Characteristics

In total, 46 patients underwent Lockdown procedures in three centers combined, but complete follow-up data were available for only 39 patients (3 females, 36 males). Seven patients were lost during follow-up (not responding to available communication channels, not attending to follow-ups). The mean age was 38.90 ± 12.68 years (range: 16–76 years). The Lockdown procedure was used as a non-primary surgical option in seven cases (two acute, five chronic). The average interval between injury and surgery was 26.69 ± 84.36 weeks (range: 0.5–510 weeks). Of the injuries, 21 (54%) were classified as Rockwood type III, 6 (15%) as type IV, and 12 (31%) as type V. The mean follow-up period was 24.51 ± 10.12 months (range: 12.8–48.9 months), as the last outpatient visit date when the functional scores were taken. The injury involved the dominant arm in 17 cases and the non-dominant arm in 15 cases (NA in 7 cases). The mean time to return to work for all patients was 7.73 ± 9.95 weeks. The duration of physiotherapy averaged 5.38 ± 6.09 weeks. Detailed results in Table 1.

#### 3.1.2. Case-Series Study, Outcomes

**Figure 2:**
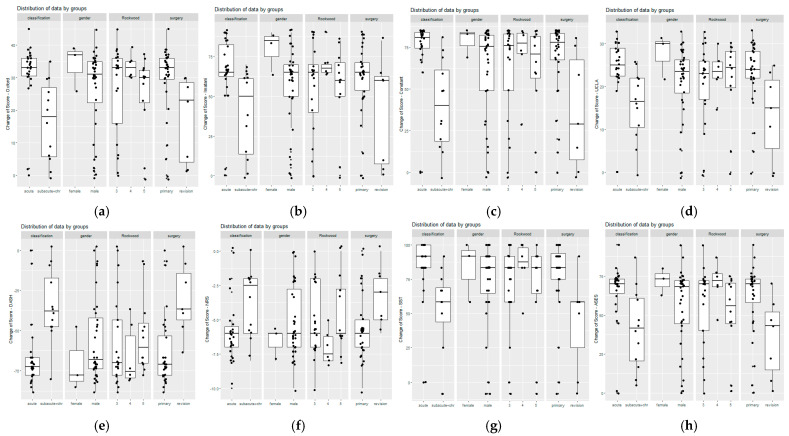
The distribution of data is shown in box plots for each score by classification in time, gender, severity by Rockwood, and previous surgery. The figures demonstrate that in all scores, acute and non-previously operated cases have better outcomes. The median and quartiles of the dataset are represented by the box plots. The horizontal line within the box represents the median, while the upper and lower quartiles are the upper and lower edges of the box. Box plots of functional outcomes: (**a**) Oxford score; (**b**) Imitani score; (**c**) Constant score; (**d**) UCLA (University of California Los Angeles Shoulder) score; (**e**) DASH (Disabilities of the Arm, Shoulder, and Hand) score; (**f**) NRS—Numerical Rating Scale; (**g**) SST (Simple Shoulder Test) score, (**h**) ASES (American Shoulder and Elbow Surgeons) score.

Significant improvements were observed in all postoperative functional scores and pain levels. The NRS decrease was in favor of both acute (mean difference: −2.26; 95% CI: −3.99–−0.53; *p* = 0.013) and primary (previously non-operated) cases (mean difference: −2.37; 95% CI: −4.42–−0.32; *p* = 0.027). The patient-reported significant visible deformity was present preoperatively in 63% and 75% of acute and chronic cases, which decreased to 33% and 8% postoperatively, but in previously operated cases there was no relevant change. The mean postoperative Oxford Shoulder Score (OSS), Constant, and Imitani scores were 44.92 ± 7.63, 90.20 ± 15.08, and 91.66 ± 16.36, respectively. Notably, OSS improved from 12.93 ± 7.14 to 44.56 ± 9.09 in acute cases, and from 29.17 ± 12.60 to 45.83 ± 2.29 in chronic cases, and the difference between the two improvements was 14.96 (95% CI: 6.45–23.47; *p* = 0.0017). Similar significant improvements were noted across all measured shoulder scores (Imitani, Constant, UCLA, DASH, SST, ASES). Table 2 presents detailed preoperative and postoperative functional outcomes for both acute and chronic cases. In all instances, the observed improvements are statistically significant, with consistently more favorable results in the acute cases (Table 2). A subgroup analysis was also conducted comparing previously non-operated cases with revision cases. The boxplots reveal notable differences across both the surgical subgroups and the acute vs. chronic cases. However, no substantial differences were observed between the subgroups with respect to gender or Rockwood classification demonstrated in Figure 2. In the non-previously operated cases, the results are similar to acute vs. chronic comparison, but the change is not significant (only relevant, according to the MCID values) in the OSS, Constant, and Imitani scores [12,18,19,20,21].

Complications were observed in 12 cases (30.8%) (in eleven patients), 7 in the acute group and 5 in the chronic group. Of these, seven cases were associated with wound redness and discharge, and four required revision surgery between two weeks and two months. Deep infection was confirmed in three cases (*S. aureus*; *C. acnes*), leading to implant removal in two cases. Thus, out of seven cases of infection, only two (5.1%) required implant removal and were considered implant failure. Of the seven cases of varying discharge, three were managed conservatively with antibiotics. No pathogen was found behind superficial (spf.) infections. Six to thirty-four months after surgery, four patients experienced re-injury after their AC reconstruction, three of whom had been treated for spf. infections. All re-injured patients underwent revision surgery with implant removal. No other implant migration was observed. A detailed table of complications is presented in Table 3.

### 3.2. Systematic Search

#### 3.2.1. Systematic Search, Basic Characteristics

**Figure 3:**
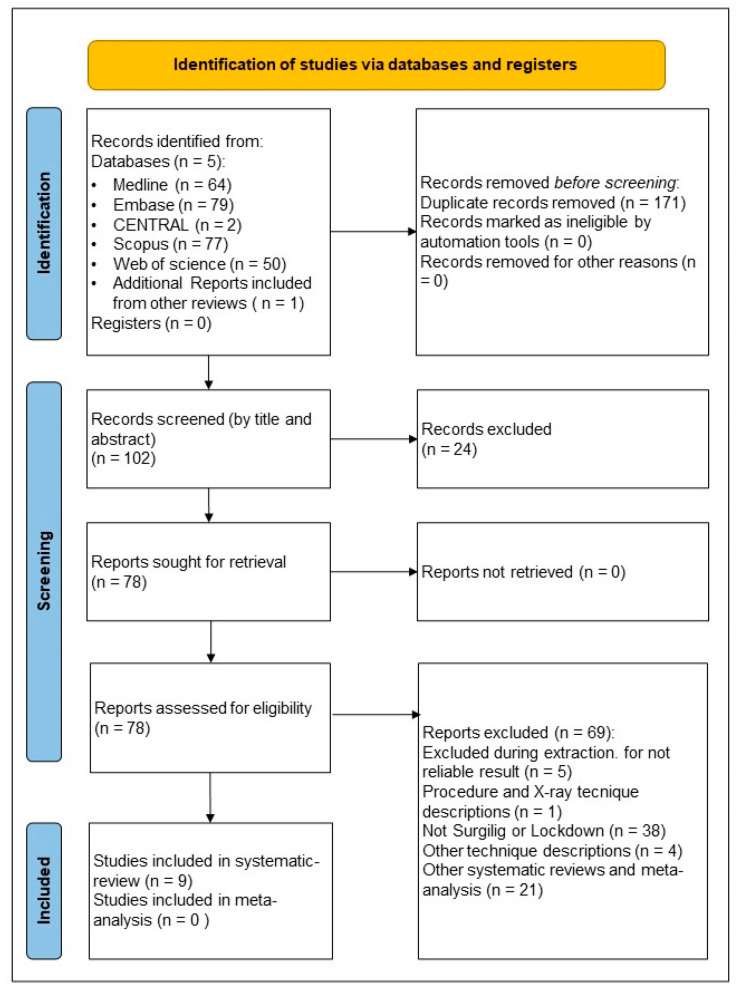
Systematic Review flowchart (Prisma 2020). In this systematic review, nine studies were evaluated from the 102 screened records after the initial search results of 273 records.

Of 273 articles, 14 met the inclusion criteria, but only 9 articles were eligible for data extraction [5,10,13,22,23,24,25,26,27,28,29,30,31,32]. These studies involved 205 patients from 2007 to 2024, with a mean age of 39.17 ± 2.96 years (range: 16–76 years). The detailed flowchart of selection by PRISMA 2020 is demonstrated in Figure 3 [5,10,13,27,28,29,30,31,32].

The mean time from injury to surgery was 27.18 ± 24.51 months (range: 0–120 months). Previous surgeries, including the Weaver–Dunn procedure and Kirschner wire fixation, were noted in some studies [13,28], but data on previous surgeries were not consistently reported. Time to return to work ranged from 5 to 6 weeks. Postoperative physiotherapy ranged from 6 to 8 weeks. The Rockwood classification of injuries was not consistently reported across studies. However, patient satisfaction exceeded 90% in four studies, with occasional pain reported in two studies; patient satisfaction, however, was not described in three studies.

#### 3.2.2. Systematic Search, Outcomes

Due to incomplete data, an accurate statistical analysis of shoulder scores was not possible. Preoperative scores were available only for OSS and Nottingham scores in three articles, whereas postoperative data were more commonly reported. The mean postoperative OSS from four articles was 38.46 ± 13.36 [5,10,29,30,32], and Constant and Imitani scores from five and two articles were 85.46 ± 4.20 [13,27,28,30,32] and 85.85 ± 6.57, respectively [13,27]. UCLA and Simple scores were only available from Carlos et al. [5]. Detailed basic characteristics are presented in Table 4. The heterogeneity of the data prevented our group from completing the meta-analysis [5,10,13,27,28,29,30,31,32]. Table 5 presents the available pre- and postoperative article numbers for each functional outcome value, reports of deformity, and pain levels.

#### 3.2.3. Systematic Search, Complications

The rate of complications rate across the studies was 34.6% (71/205 patients), with the most common issues being implant-related problems such as proud metalwork (a noticeable, protruding bulk of metalwork beneath the skin), loosening, stiffness, joint subluxation, and subacromial impingement, occurring in 30.7% of cases. Spf. infection and discharge occurred in eight cases. Reoperations were performed in 15 cases (7.3%), and 11 (5%) implants were removed due to re-injury, subacromial impingement, and infection. The cases were considered as implant failures that had to be removed during revisions due to non-re-injury. Therefore, the aggregated implant failure rate was 11/205 (5.4%). A detailed list of complications in the SR is in Table 6.

#### 3.2.4. Systematic Search, Risk of Bias Assessment

The result of the risk of bias assessment is presented in Table S3, Figures S1 and S2 in the Supplementary Material, which shows a low level of evidence due to their study designs.

## 4. Discussion

Our study aimed to evaluate clinical outcomes following Lockdown surgery. It was also noted that although over 150 different surgical methods had been described for Rockwood III injuries or higher, the gold standard remained a matter of debate due to the lack of high-quality randomized trials and heterogeneity of studies, which is approved by our systematic literature review as well [6,33].

One of the earliest methods for AC reconstruction, Kirschner or K-wire fixation, has been diversely affected by severe complications such as wire migration with nerve and vascular damage, for example [34]. Alternative methods, such as the Bosworth screw (with or without K-wire augmentation), are not free from complications due to the rigid nature of the repair of a dynamic structure [35]. Iterations of hook plate fixation provide direct AC joint stabilization but require a second surgery to remove the implant. The method is known to have metalwork-related complications as well as AC joint arthritis, subacromial impingement, and erosion of the acromion [36,37]. The complication rate for hook plate stabilization has been reported to be 26.3%, with shoulder stiffness and pain on elevation being the most common issues [35,38].

The Weaver–Dunn procedure (1972) is a non-anatomical repair and is known for its low repair stability. The repair has been shown to be 70% weaker than native ligaments, resulting in a high rate of recurrent deformity and a high reoperation rate [39,40,41].

Recently, newer, more anatomical surgical procedures have become increasingly popular [39,40]. The Lockdown artificial ligament allows anatomical reconstruction, with a pull-out strength of 1700 N compared to 483 N–500 N of CC sling from Fiberwire [10]. The synthetic graft promotes favorable tissue reactions and ingrowth, contributing to the strength and flexibility of the reconstructed system [9]. But complications were also reported, such as coracoid or clavicle fracture, soft tissue reactions, or redislocations [33].

In our cohort, functional outcomes were significantly better in the acute and most scores of the non-previously operated group (as demonstrated in Table 2). Although MCIDs have not been established for AC joint dislocations, thresholds from rotator cuff pathology, as cited by Lindborg et al., provide a reference [12]. In the subgroup comparison between primary and revision cases, most functional outcome differences were statistically significant, except for the OSS, Constant, and Imitani scores. Nonetheless, these results exceeded established MCID thresholds, indicating clinical relevance. Specifically, mean differences were 12.22 (95% CI: −0.34–24.78; *p* = 0.051) for OSS and 32.07 (95% CI: −0.73–64.86; *p* = 0.054) for the Constant score, both exceeding the MCIDs of 3.3 and 10 [18,42]. No MCID has been established for the Imitani score in the literature. A sample size of 71 is needed for 80% power, but the MCID and the visualization by box plots confirm better results in the non-previously operated group.

Our postoperative results for the Lockdown cohort were similar to those in the literature. Combined figures from the literature show the following postoperative scores. 38.46 ± 13.36 (OSS), 85.46 ± 4.20 (Constant), and 85.85 ± 6.57 (Imitani), with our cohort study showing similar results: 44.92 ± 7.63, 90.20 ± 15.08, and 91.66 ± 16.36, respectively. In their systematic reviews, Sircana and Borbas et al. reported a mean postoperative Constant score between 87.2 and 90.6, which is in close agreement with our findings [43,44]. Kumar et al. also observed positive Nottingham score trends, paralleling our improvements [29]. However, the lack of data on preoperative scores across other studies limits direct comparisons, as most of the literature focuses solely on postoperative functional scores. Narang et al. and Kumar et al. found that Lockdown outperformed the Weaver–Dunn procedure in terms of functional scores and recovery time [10,29].

Complication differences (Table 7) provide a detailed comparison of complications in our Lockdown cohort vs. the literature. Our analysis (Table 3) showed a similar complication rate (30.8% vs. 34.6%), with infections notably more frequent in our cohort (17.9% vs. 3.9%). Three infections were managed with antibiotics, while two deep infections (5.1%) required implant removal, comparable to the literature’s data.

The exclusive postoperative follow-up by surgical teams in Hungary may contribute to the higher detection of spf. infections. Two of the seven infected cases were revisions from other surgical methods, and our cohort had more non-primary Lockdown cases than the literature cohort. No bacterial culture results were reported in the reviewed studies. Re-injury rates in our cohort were significantly higher (10.3% vs. 1.5%), likely due to mixed patient cases, including at least three high-energy traumas capable of damaging native AC joints. Other observed complications included proud metalwork (7.7%) and implant loosening (5.1%), but no stiffness, subluxation, or subacromial impingement. Literature data report subluxation (16.1%), proud metalwork (7.3%), implant loosening (3.9%), stiffness (1.5%), subacromial impingement (1.5%), and graft rupture (0.4%). Implant removal rates were higher in our cohort (15.4% vs. 5.4%), primarily due to re-injury, deep infections (cases not responding to antibiotics), and a higher percentage of cases revised from previous unsuccessful operations. The “true” implant failure rate, accounting for infections and implant loosening without re-injury, was similar in our cohort and in the literature (5.1% vs. 5.4%). The reoperation rate in our study (20.5%) exceeded reported rates (7.3%), mainly due to re-injuries and deep infections (*S. aureus*). Causes of reoperation in the literature included subacromial bursitis (three cases), proud metalwork (two cases), infections (seven cases), implant loosening (one case), and graft rupture (one case). In the comparison between the modified Weaver–Dunn procedure and Lockdown, the latter had less persistent pain postoperatively, but the rate of spf. infection was similar [29]. Saraglis et al. (n = 48) reported a higher infection rate in the double endobutton group (three vs. zero cases), whereas radiological failure was lower in the Lockdown group (four vs. six cases) [30]. SR-s have shown overall complication rates of 9.1–14.9% for synthetic systems, compared to 15.5–22% for biological grafts, 29.7% for internal fixations, and 17.3% for ligament transfers [43,44]. Chronic AC joint instability fixation and revision procedures are underreported, as noted by Berthold et al. and evidenced in Table 6, where four out of nine studies provide insufficient detail [45]. This limitation may affect result interpretation. For all AC joint reconstruction techniques, the overall complication rate is 20.8%, and 27% in CC ligament reconstruction, according to the meta-analyses by Gowd et al. and Martetschlager et al. [46,47,48]. In addition, the issue with rigid fixation devices is the loss of motion, which increases the probability of breakage, implant loosening, subacromial erosions, impingements, and additional operation for implant removal.

Detailed data on the duration of physiotherapy, postoperative deformity, and timing of return to work were frequently unavailable [29,30,49]. Additionally, inconsistent pain reported in the literature hindered accurate comparisons [5,10,13,27,28,29,30,31]. The design of the study did not allow the evaluation of possible positive effects of time elapsed, physiotherapy, and medication on the outcome of surgery.

The literature frequently compares conservative and surgical approaches to AC joint injuries. Boström et al. (2022; 124 cases) found no significant differences between Hook plate fixation and conservative therapy in Rockwood type III and V AC joint injuries [50]. The 2015 randomized controlled trial of the Canadian Orthopaedic Trauma Society (76 cases) also compared Hook plate fixation to nonoperative treatment in Rockwood >III injuries. They reported better short-term functional results in the nonoperative group, but no significant long-term differences [51]. Similarly, Murray et al. (2018; 60 cases) found no differences in functional outcomes at one year between open reduction with suspensory device fixation (ORTSD) and conservative therapy in Rockwood type III and IV AC joint injuries [52]. Although the conservative group had better OSS scores at six weeks, many required delayed surgery due to dissatisfaction [52]. A multicenter randomized trial by Tauber et al. (85 cases) found no significant differences between surgical (tightrope, Hook plate) and nonsurgical management in Rockwood III injuries, although surgical treatment had slower recovery, higher complication rates, and increased posttraumatic osteoarthritis and heterotopic ossifications [53].

While speculative, our clinical experience suggests poor inter-assessor agreement in diagnosing Rockwood III and higher injuries. Evidence supports non-operative treatment for true Rockwood III cases, yet some initially diagnosed patients remain symptomatic. Identifying a reliable surgical bailout method for these chronic and post-failure cases is essential.

### 4.1. Strength and Limitation

Our study presents several strengths, particularly in the comprehensive evaluation of the Lockdown procedure for AC joint dislocations, along with an up-to-date review of the literature. However, it has some limitations. The absence of control groups restricts direct comparison with other surgical methods. While this is a significant limitation, similar study designs are common in the literature. Additional sources of bias include selection bias, due to the lack of total number of ACJ dislocations treated at these centers, and observer bias, resulting from the lack of blinding and the subjective nature of surgeons’ assessments.

The sample size, though moderate, is still significant, given the systematic search we conducted, adding value to our findings. Selection bias is another limitation, but we mitigated this by combining prospective data with prospective follow-up, increasing the reliability of our results. The average follow-up period of two years is adequate for short-term outcomes, but longer follow-up is needed to understand long-term effects. We were unable to conduct a meta-analysis due to the heterogeneity in the included studies, therefore we conducted a systematic review, which highlights the need for further research.

### 4.2. Implications for Research and Practice

The rapid integration of results into clinical practice is beneficial [54,55]. In clinical practice, the Lockdown technique can be recommended for acute AC joint reconstruction, with a complication rate comparable to other methods. We observed a mild complication rate in patients who underwent Lockdown revision due to a previously failed surgery. Further research with robust study designs and reliable data is needed to compare different reconstruction methods, such as Lockdown versus other suspension techniques. Particularly pressing is the lack of data on patients who have previously undergone non-Lockdown surgery and the revision to Lockdown.

## 5. Conclusions

Our study suggests that the Lockdown technique is effective for primary and acute AC joint injuries. A relatively high complication rate was observed across various surgical methods, which may indicate that the anatomical location is inherently prone to complications.

## Figures and Tables

**Table 1:** Basic characteristics of the case series.

Basic Characteristics		All Patient	Time		Surgery	
			Acute <3 Weeks	Chronic >3 Weeks	Primary (Non-Previously Operated)	Non-Primary Operation (Revision)
No. of patients		39	27	12	32	7
Primary/Revision		32/7	25/2	7/5	-	-
Acute/chronic			-	-	25/7	2/5
Gender (M/F)		36/3	25/2	11/1	29/3	7/0
Mean age at op. (years)		38.90 ± 12.68	35.52 ± 9.46	46.5 ± 15.2	36.69 ± 12.08	36.91 ± 14.33
Mean time of interval (injury—op) (weeks)		26.69 ± 84.36	1.07 ± 0.65	84.33 ± 139.11	25.44 ± 87.90	23.62 ± 87.86
Mean time to return to work		7.73 ± 9.95	6.87 ± 10.49	9.66 ± 8.73	8.57 ± 11.68	8.84 ± 11.73
Follow-up (months)		24.51 ± 10.12	23.18 ± 8.98	27.53 ± 12.19	23.84 ± 10.45	22.76 ± 9.72
Rockwood classification:						
	III	21 (54%)	15 (55%)	6 (50%)	18 (56%)	3 (43%)
	IV	6 (15%)	4 (15%)	2 (17%)	5 (16%)	1 (14%)
	V	12 (31%)	8 (30%)	4 (23%)	9 (28%)	3 (43%)

Table of basic characteristics of the case series.

**Table 2:** Case series, changes in scores.

	Acute				Chronic				Difference				*p* Value
	Preop		Postop		Preop		Postop		Acute		Chronic		(diff. Acute -diff.chr)
	Mean	SD	Mean	SD	Mean	SD	Mean	SD	Mean	SD	Mean	SD	
OSS	12.93	±7.14	44.56	±9.08	29.11	±12.60	45.83	±2.29	−31.63	±9.62	−16.67	±12.42	0.0017
IMITANI	26.11	±16.78	91.67	±19.22	52.08	±30.41	91.67	±9.13	−65.56	±22.12	−39.58	±27.26	0.0096
CONSTANT	18.22	±15.37	91.11	±17.33	47.83	±25.66	88.17	±9.53	−72.89	±22.07	−40.33	±26.96	0.0017
UCLA	8.52	±4.85	32.59	±6.20	17.00	±6.86	32.58	±3.63	−24.07	±7.78	−15.58	±8.27	0.0069
DASH	73.64	±16.35	7.65	±17.41	41.90	±21.55	6.97	±8.69	65.98	±20.24	34.93	±22.85	0.0004
SST (%)	6.47	±17.51	88.59	±19.65	34.74	±27.72	86.12	±10.26	−82.12	±25.91	−51.38	±32.54	0.0099
ASES	26.63	±15.07	90.44	±20.09	52.08	±26.12	93.25	±6.55	−63.81	±20.83	−41.17	±25.67	0.0151
NRS (1–10)	6.78	±1.76	0.85	±2.38	4.58	±2.81	0.92	±0.94	5.93	±2.34	3.66	±2.42	0.0131

Table of pre-and postoperative scores in acute and chronic cases with significant differences (*p* < 0.05). (diff.: difference).

**Table 3:** Case series complications.

	No. of Group	Patient ID	Previous Surgery	Proud Metalwork	Implant Loosening	Discharge	Trauma	Reoperation	Reason of Reoperation	Time Until Reoperation (Days)	Lockdown Implant Extracted	Result of Bacteriogram
Acute	27		1	2	1	4	3	5		525.6 ± 613.48	4	
		H1	1	0	0	1	0	1	discharge	18	1	*S. aureus*
		H7	0	0	1	1	1	1	trauma	1045	1	0
		H8	0	0	0	1	0	1	discharge	58	0	0
		H27	0	0	0	0	1	1	trauma	1326	1	0
		P4	0	0	0	1	1	1	trauma	181	1	*S. aureus*
		H13	0	1	0	0	0	0	0	0	0	0
		H22	0	1	0	0	0	0	0	0	0	0
Chronic	12		1	1	1	3	1	3		141.33 ± 216.23	2	
		O3	0	0	1	1	0	1	discharge	19	1	*C. acnes*
		O4	1	0	0	1	1	1	trauma	391	1	0
		P5	0	0	0	1	0	1	discharge	14	1	*S. aureus*
		O2	0	1	0	0	0	0	0	0	0	0

Detailed table of complications in our case series.

**Table 4:** Basic characteristics of the systematic search.

	Year of Publication	Origin of Authors	No. of Cases	Study Design	Male	Female	Mean Age (Year)	Rockwood Classification	Mean Interval (Months)	Mean Follow Up (Months)
Narang et al. [10]	2023	UK	42	RCS	39	3	42.2	8:III; 4:IV; 30:V;	71.1	68
Jeon et al. [13]	2007	South Korea	11	RCS	11	0	39	9:III; 1: IV; 1:V;	18	55
Bhattacharya et al. [27]	2008	UK	11	PCS	10	1	35.1	>III	21	24
Wood et al. [31]	2009	UK	10	RCS	NA	NA	NA	>III	NA	6
Carlos et al. [5]	2011	UK	45	PCS	32	13	37.6	>III	7.2	26.9
Cetinkaya et al. [28]	2018	Turkey	16	RCS	16	0	38.5	>III	NA	20
Kumar et al. [29]	2014	UK	24	RCS	NA	NA	42	13:III; 4:IV; 7:V;	39	30
Saraglis et al. [30]	2022	UK	25	RCS	22	3	36	IV: 19; V:6	NA	NA
Wright et al. [32]	2015	UK	21	RCS	21	0	43	12:III; 1:IV; 8:V;	6.80	30
No. of patients			205							
No. of studies	9									

Basic characteristics of the systematic search. (RCS: retrospective cohort study; PCS: prospective cohort study).

**Table 5:** Heterogeneity of the literature.

	**Oxford**	**ASES**	**Nottingham**	**Imitani**	**Constant**
Preop.	2	0	1	0	0
Postop.	5	0	1	2	5
	**UCLA**	**Simple**	**Pain (NRS**)	**Deformity**	**Complications**
Preop.	0	0	2	1	
Postop.	1	1	4	0	8

Summary of data collected from the articles. Presentation of the heterogeneity of the literature. (ASES: American Shoulder and Elbow Shoulder score; UCLA: University of California Los Angeles Shoulder Score; NRS: Numerical Rating scale).

**Table 6:** Complications in the systematic review.

Article	Number of Patients	Implant Loosening, Osteolysis	Subluxation	Other (Stiffness, Graft Rupture, Subacromial Impingement)	Proud/Prominence Metalwork	Superficial Infection, Discharge	Trauma	Previous Surgery	# of Reoperation	Reoperation Due to Non Re-Injury	Reasons for Reop	Previous Surgery	Time Until Reoperation (Months)	Lockdown Implant Extracted	Result of Bacteriogram
Narang et al. [10]	42	0	8 (X-ray)	3 (stiffness)	5 (proud)	1	0	0	2	2	Proud metal.	NA	NA	2	NA
Jeon et al. [13]	11	1 (loosening)	11 (X-ray)	2 (sub)	0	0	1	3 (Weaver Dunn)	2	2	Subacr. Burs.	0	6–10	1	NA
Bhattacharya et al. [27]	11	4 (lysis)	0	1 (graft)	4 (prom.)	0	NA	0	1	1	Graft rupture	0	6	1	NA
Wood et al. [31]	10	0	0	0	0	0	0	NA	0	0	0	0	0	0	0
Carlos et al. [5]	45	1 (loosenin)g	13 migration = sublux (X-ray)	0	6 (skin irritation)	NA	0	NA	7	7	6 inf., 1 loosening	NA	9	6	0
Cetinkaya et al. [28]	16	1 (lysis)	1 (X-ray)	0	0	0	0	1 (K-wire)	0	0	0	0	0	0	0
Kumar et al. [29]	24	0	0	0	0	4	1	NA	1	0	0	0	NA	0	0
Saraglis et al. [30]	25	1 (osteolysis)	0	0	0	3	0	0	1	1	infection	0	NA	1	0
Wright et al. [32]	21	0	0	1 (subacrom. imp.)	0	0	1	NA	1	1	Subacr. Burs.	0	NA	0	0
	205	8	33	7	15	8	3	4	15	14			6–10	11	0
	100%	3.9%	16.1%	3.4%	7.3%	3.9%	1.5%	2.0%	7.3%	6.8%				5%	

Detailed table of complications in the literature. (NA: not available; #: number; prom: prominent).

**Table 7:** Comparison of complications.

Complication Differences				
	Our Results		Sys Search	
No. of patients	39		205	
No. of complications (without trauma)	12	30.8%	71	34.6%
Time interval (from injury to surgery, weeks)	26.69 ± 84.36 (0.5–510)		108.72 ± 98.04 (27.2–284.4)	
Stiffness, subacromial imp, graft failure	0	0.0%	7	3.4%
Subluxation	0	0.0%	33	16.1%
Proud metalwork, graft rupture, bursitis, stiffness	3	7.7%	15	7.3%
Implant loose	2	5.1%	8	3.9%
Spf. infection	7	17.9%	8	3.9%
Re-injury	4	10.3%	3	1.5%
Previous surgery	7	17.9%	4	2.0%
Re-op.	8	20.5%	15	7.3%
Implant remove	6	15.4%	11	5.4%
Implant failure	2	5.1%	11	5.4%

Comparison of complications in the literature and in our case series. (Spf.: superficial, Sys: systematic).

## Data Availability

For further studies, our reported results can be provided to other authors by making contact with the corresponding author’s email address.

## Supplementary Materials

The following supporting information can be downloaded at: https://www.mdpi.com/article/10.3390/jcm14124046/s1.

## Abbreviations

The following abbreviations are used in this manuscript:

AC	acromioclavicular
CC	coracoclavicular
OSS	Oxford shoulder score
LD	Linear dichroism
ORTSD	open reduction and suspensory device fixation
UCLA	University of California-Los Angeles shoulder score
ASES	American shoulder and elbow surgeons
SST	simple shoulder test
DASH	disabilities of the arm, shoulder, and hand
NRS	numerical Rating Scale
vs.	versus
spf.	superficial
SR	systematic review
MA	meta-analysis
K-wire	Kirschner wire
MCID	minimal clinically important difference
NA	not available
