# Supplementary material for: Acromioclavicular Reconstruction Using the Lockdown Technique: A Case Series and Systematic Review
Source: J Clin Med. 2025 Jun 7;14(12):4046. doi: 10.3390/jcm14124046 (PMC12194664; doi:10.3390/jcm14124046)
Supplement: Supplementary file 1 [file jcm-14-04046-s001.zip › Table_S3_RiskofBias.pdf]

TABLE_S3. RISK OF BIAS

RISK OF BIAS	Q1	Q2	Q3	Q4	Q5	Q6	Q7	Q8	Q9	Q10
NARANG										
Jeon										
Bhattacharya										
Wood										
Carlos										
Cetinkaya										
Kumar										
Saraglis										
Wright										

Q1	Were there clear criteria for inclusion in the case series?
Q2	Was the condition measured in a standard, reliable way for all participants included in the case series?
Q3	Were valid methods used for identification of the condition for all participants included in the case series?
Q4	Did the case series have consecutive inclusion of participants?
Q5	Did the case series have complete inclusion of participants?
Q6	Was there clear reporting of the demographics of the participants in the study?
Q7	Was there clear reporting of clinical information of the participants?
Q8	Were the outcomes or follow-up results of cases clearly reported?
Q9	Was there clear reporting of the presenting site(s)/clinic(s) demographic information?
Q10	Was statistical analysis appropriate?

Table of the risk of bias assessment.
