# Supplementary figures and images for: Acromioclavicular Reconstruction Using the Lockdown Technique: A Case Series and Systematic Review
Source: J Clin Med. 2025 Jun 7;14(12):4046. doi: 10.3390/jcm14124046 (PMC12194664; doi:10.3390/jcm14124046)

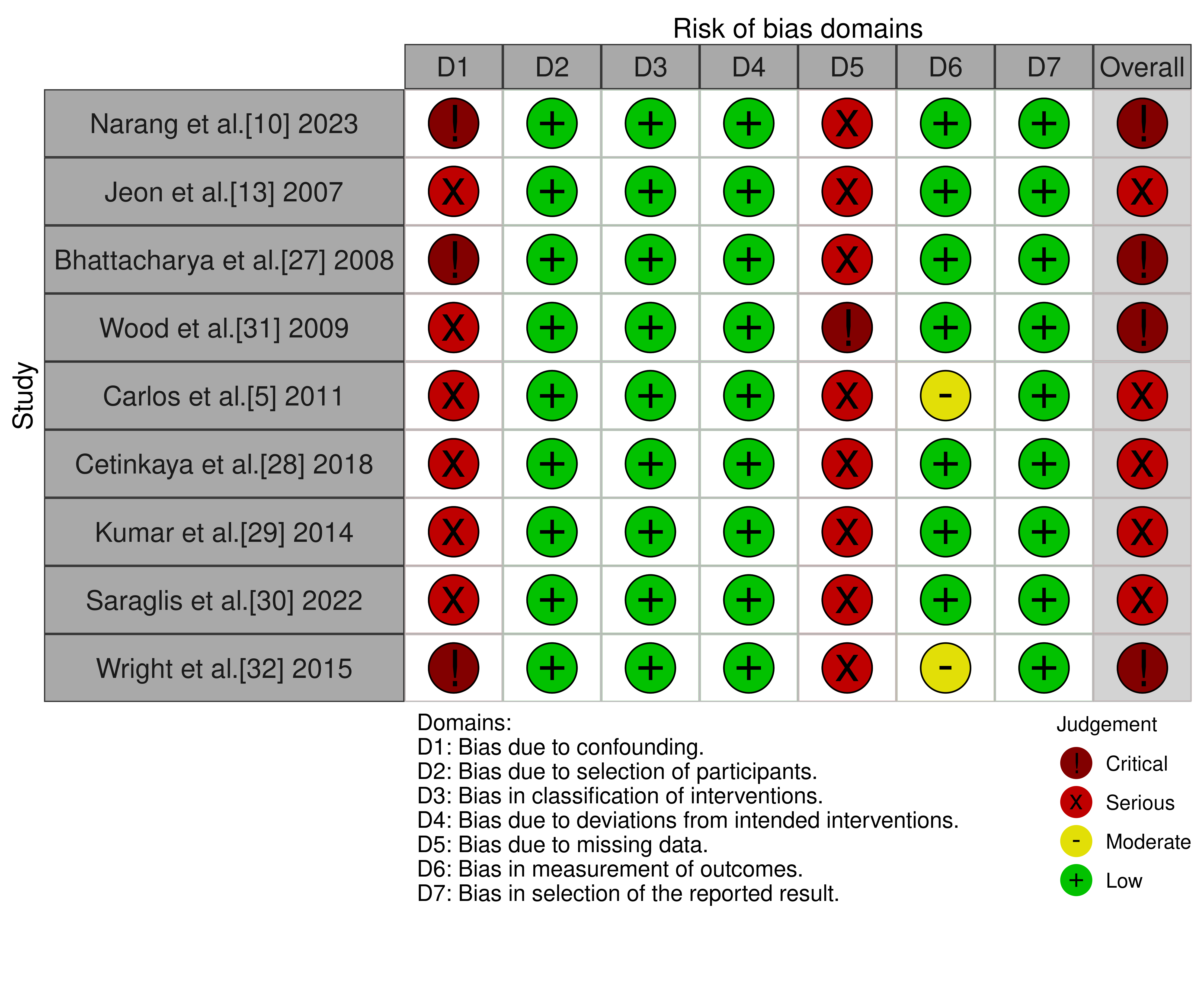

Supplement: Supplementary file 1 [file jcm-14-04046-s001.zip › Figure_S1_ROBINS-I .png]

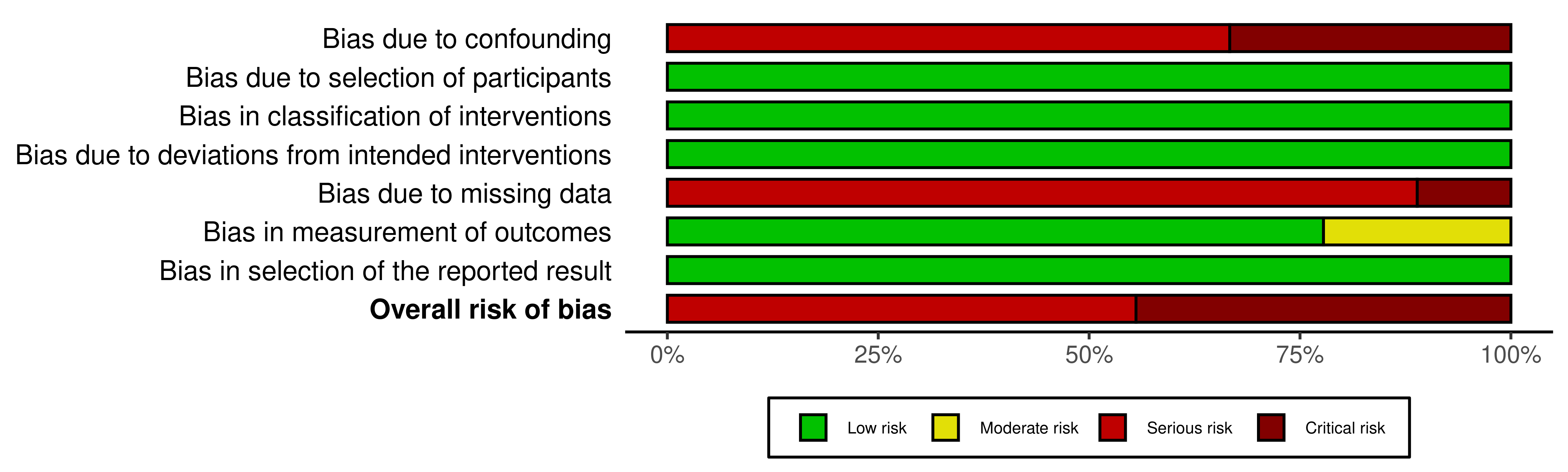

Supplement: Supplementary file 1 [file jcm-14-04046-s001.zip › Figure_S2_ROBINS-I weighted.png]
